# Supplementary material for: Pinocembrin Protects from AGE-Induced Cytotoxicity and Inhibits Non-Enzymatic Glycation in Human Insulin
Source: Cells. 2019 Apr 26;8(5):385. doi: 10.3390/cells8050385 (PMC6562854; doi:10.3390/cells8050385)
Supplement: Supplementary file 1 [file cells-08-00385-s001.pdf]

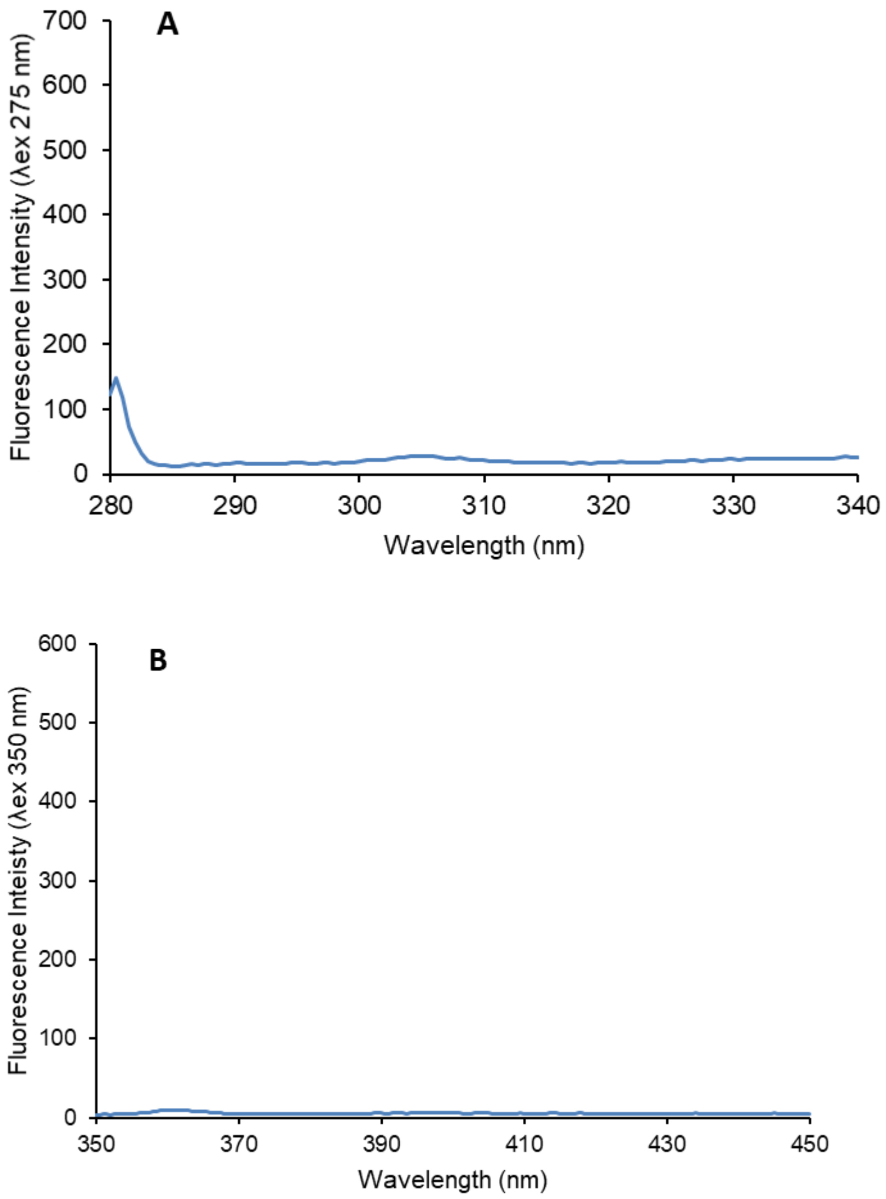

Emission spectra of pinocembrin in phosphate buffer 20 mM pH 7.0. (A) Excitation wavelength 275 nm, Pinocembrin concentration 40 μM. (B) Excitation wavelength 350 nm, Pinocembrin concentration 20 mM.
